# Surgical Operations in the Franco-German War

**Published:** 1873-03-15

**Authors:** Edmund Andrews

**Affiliations:** Professor of Surgery in Chicago Med. College; No. 6 Sixteenth street, Chicago


### SURGICAL OPERATIONS IN THE FRANCO-GERMAN WAR.

COLLATED BY EDMUND ANDREWS, M.D., PROFESSOR OF SURGERY IN CHICAGO MED. COLLEGE.

Dr. Carl Prœgler, late Staff Surgeon in the American Ambulance, attached to the Prussian army, and now residing at Addison, Dupage county, Ill., has been kind enough to place in my hands a considerable collection of his recorded observations. He first served, as above stated, in the Prussian army, but, at the close of the war, went to Paris, and served with the French forces engaged in besieging the Communists. Here, though a non-combatant, and serving their own sick and wounded, his life was in considerable danger, from the fact that he was a German by birth, and liable, if his nationality was discovered, to be assassinated by some Frenchman, maddened by defeat. He succeeded in escaping, by passing as an Englishman, and talking only the English and French languages. As these records are among the earliest accessible results of the surgery of that great war, they will possess an interest for the readers of THE EXAMINER, although they are less perfect than they otherwise would be, from the fact that an ambulance corps does not keep its patients from the day of their being wounded to their recovery or death, but must often transfer them to general hospitals, whenever the order comes to march.

The following table shows the results of over two hundred large operations. In this table the term *primary*, applied to operations, signifies that they were performed within twenty-four hours of the injury; *intermediate*, that it was done after twenty-four hours, and during the period of acute inflammation; while *secondary* means after the acute stage has passed.

### TABLE I.

OPERATIONS ARRANGED BY LOCATION AND TIME OF PERFORMANCE.

| | PRIMARY. | | INTERMEDIATE. | | SECONDARY. | | TOTAL. | |
|---|---|---|---|---|---|---|---|---|
| | REC'D | DIED | RECOVERED. | DIED. | RECOVERED | DIED. | REC'D | DIED. |
| Amp. at hip-joint (disarticulation) time not stated | ---- | ---- | ---- | ---- | ---- | ---- | 0 | 1 |
| Amp. at upper third of fermur | 2 | 2 | 1 | 0 | 15 | 4 | 18 | 7 |
| " middle third of fermur | 4 | 7 | 0 | 1 | 14 | 2 | 18 | 10 |
| " lower third of fermur | 4 | 3 | 4 | 2 | 30 | 12 | 38 | 17 |
| " knee-joint | 0 | 0 | 0 | 0 | 4 | 0 | 4 | 0 |
| " upper third of leg | 2 | 1 | 4 | 3 | 1 | 1 | 7 | 5 |
| " middle third of leg, time not stated | ---- | ---- | ---- | ---- | ---- | ---- | 9 | 1 |
| " lower third of leg | 1 | 0 | 0 | 0 | 6 | 4 | 7 | 4 |
| " ankle (Pirogoff's) | 0 | 0 | 0 | 0 | 1 | 2 | 1 | 2 |
| " " (Syme's) | 0 | 0 | 0 | 0 | 1 | 1 | 1 | 1 |
| " through tarsus and matatarsus | 0 | 0 | 0 | 0 | 11 | 0 | 11 | 0 |
| " arm, time not stated | ---- | ---- | ---- | ---- | ---- | ---- | 4 | 0 |
| " forearm, time not stated | ---- | ---- | ---- | ---- | ---- | ---- | 1 | 1 |
| Excision of knee-joint, time not stated | ---- | ---- | ---- | ---- | ---- | ---- | 1 | 34 |
| " " patella | 0 | 0 | 0 | 0 | 0 | 3 | 0 | 3 |
| " " head of radius | 1 | 0 | 0 | 0 | 0 | 0 | 1 | 0 |
| " " entire elbow | 1 | 0 | 0 | 0 | 0 | 0 | 1 | 0 |
| Totals | 15 | 13 | 9 | 6 | 83 | 29 | 122 | 86 |

It will be observed that in this table the mortality of the amputations of the femur is only about half as much at the upper third as at the middle and lower thirds, thus completely inverting the usual rule. I am unable to account for this anomaly, but I presume it is through some error in that portion of the statistics furnished to Dr. Prœgler by other officers, as he is very accurate in all his own observations.

The frightful mortality of the excisions of the knee-joint will perhaps astonish the civil surgeon, only one patient surviving out of thirty-five excisions. This, however, is corroborated by the experience of all military surgeons. In the American war the mortality of excisions of the knee, though not quite so great as that in the table, was still terrible, being fully 77 per cent. (twenty deaths out of twenty-six excisions). The fact is, that conservative surgery in gunshot wounds of the knee is wholly wrong. All such cases should be amputated on the field of battle, unless there exists some complication forbidding the operation.

The following additional cases were observed by Dr. Prœgler, in the Bavarian army, at the Receiving Hospital No. 8, after the battles at Ausang and Massy, in France, in the autumn of 1870. As this was only a Receiving Hospital, many of the cases were sent away before they were terminated; hence I am unable in most cases to deduce from the record the proportion of deaths and recoveries after each injury and operation, but the following statistical facts are important.

The total number of wounded received at this hospital was 361, of whom nine were officers, and 352 non-commissioned officers and privates. The classification of the injuries is as follows:

| | |
|---|---|
| Gunshot wounds | 349 |
| Sabre " | 2 |
| Lance " | 3 |
| Other injuries | 7 |

This shows how completely firearms have superseded the sword in modern warfare.

Table II shows the very great proportion of cases which died of pyæmia, and the period of time which elapsed between the wound and the death.

### TABLE II.

TIME AND NUMBER OF DEATHS FROM PYÆMIA, TETANUS, AND OTHER CAUSES.

| NUMBER OF DAYS FROM WOUND. | PYÆMIA, AND OTHER SEPTIC DISEASES. | TETANUS. | OTHER CAUSES. | NUMBER OF DAYS FROM WOUND. | PYÆMIA, AND OTHER SEPTIC DISEASES. | TETANUS. | OTHER CAUSES. |
|---|---|---|---|---|---|---|---|
| | | | | | Bro't forw'd 11 | 5 | 19 |
| 1 | -- | -- | 2 | 26 | 1 | -- | -- |
| 2 | -- | 1 | 7 | 27 | -- | 1 | -- |
| 3 | -- | -- | 1 | 28 | -- | -- | 1 |
| 6 | 1 | -- | -- | 29 | 1 | -- | -- |
| 7 | -- | 1 | -- | 30 | -- | -- | 1 |
| 9 | 1 | -- | -- | 33 | 1 | -- | -- |
| 10 | -- | -- | 1 | 35 | 1 | -- | -- |
| 11 | -- | 2 | 3 | 38 | 1 | -- | -- |
| 12 | 1 | -- | 1 | 40 | 2 | -- | -- |
| 13 | 1 | -- | 1 | 42 | 1 | -- | -- |
| 14 | 4 | -- | -- | 43 | 1 | -- | -- |
| 15 | 1 | -- | 1 | 44 | 1 | -- | -- |
| 17 | -- | -- | 1 | 48 | 1 | -- | -- |
| 22 | -- | -- | 1 | 50 | 1 | -- | -- |
| 23 | -- | 1 | -- | 52 | 1 | -- | -- |
| 24 | 2 | -- | -- | -- | -- | -- | -- |
| | Car'd forw'd 11 | 5 | 19 | Total | 24 | 6 | 21 |

It appears, therefore, that the septic diseases produced an enormous mortality — nearly fifty per cent. of all the deaths. Billroth, in his letters from the same war, says that, in the hospital at Weissenburg, twenty-nine deaths out of thirty-five, or eighty-three per cent., were from pyæmia, and gives a sort of half-way opinion that pure air has not so much to do with preventing the scourge as is commonly supposed. Experience in the American war, however, showed that wounded men, treated in the open air, rarely had this trouble. I think, therefore, that Prof. Billroth, in spite of his eminence, is mistaken, and the more so because the ideas of what constitutes pure air in Central and Eastern Europe are far behind those of America. The nervous dread of "catching cold" is there carried to what seems in our eyes a ludicrous excess; and it is almost impossible for the surgeon, dealing with soldiers, and nurses imbued from childhood with the fear of external air, to secure full obedience to orders given with the view of promoting free ventilation of the wards. The reader will observe that Dr. Prœgler's mortality from pyæmia was considerably less than Prof. Billroth's, showing his superior attention to ventilation.

It will be seen that the deaths from pyæmia do not commence until the sixth day; that they are most numerous from the tenth to the fifteenth days; but continue in a scattering way until the fifty-second. Tetanus follows a similar law; while the deaths from other causes are most abundant on the first and second days (doubtless from the prevalence of shock), but grow less and less frequent, until they almost cease after the twenty-second day.

No. 6 Sixteenth street, Chicago.

